# Enhancing Frailty Assessments for Transcatheter Aortic Valve Replacement Patients Using Structured and Unstructured Data: Real-World Evidence Study

**DOI:** 10.2196/58980

**Published:** 2024-11-27

**Authors:** Mamoun T Mardini, Chen Bai, Anthony A Bavry, Ahmed Zaghloul, R David Anderson, Catherine E Crenshaw Price, Mohammad A Z Al-Ani

**Affiliations:** 1 Health Outcomes and Biomedical Informatics, College of Medicine, University of Florida, Gainesville, FL, United States; 2 Department of Internal Medicine, University of Texas Southwestern Medical Center, Dallas, TX, United States; 3 Division of Cardiovascular Medicine, College of Medicine, University of Florida, Gainesville, FL, United States; 4 Department of Clinical and Health Psychology, College of Public Health and Health Professions, University of Florida, Gainesville, FL, United States; 5 Perioperative Cognitive Anesthesia Network for Alzheimer’s Disease and Related Dementias, University of Florida, Gainesville, FL, United States

**Keywords:** transcatheter aortic valve replacement, frailty, cardiology, machine learning, TAVR, minimally invasive surgery, cardiac surgery, real-world data, topic modeling, clinical notes, electronic health record, EHR

## Abstract

**Background:**

Transcatheter aortic valve replacement (TAVR) is a commonly used treatment for severe aortic stenosis. As degenerative aortic stenosis is primarily a disease afflicting older adults, a frailty assessment is essential to patient selection and optimal periprocedural outcomes.

**Objective:**

This study aimed to enhance frailty assessments of TAVR candidates by integrating real-world structured and unstructured data.

**Methods:**

This study analyzed data from 14,000 patients between January 2018 and December 2019 to assess frailty in TAVR patients at the University of Florida. Frailty was identified using the Fried criteria, which includes weight loss, exhaustion, walking speed, grip strength, and physical activity. Latent Dirichlet allocation for topic modeling and Extreme Gradient Boosting for frailty prediction were applied to unstructured clinical notes and structured electronic health record (EHR) data. We also used least absolute shrinkage and selection operator regression for feature selection. Model performance was rigorously evaluated using nested cross-validation, ensuring the generalizability of the findings.

**Results:**

Model performance was significantly improved by combining unstructured clinical notes with structured EHR data, achieving an area under the receiver operating characteristic curve of 0.82 (SD 0.07), which surpassed the EHR-only model’s area under the receiver operating characteristic curve of 0.64 (SD 0.08). The Shapley Additive Explanations analysis found that congestive heart failure management, back problems, and atrial fibrillation were the top frailty predictors. Additionally, the latent Dirichlet allocation topic modeling identified 7 key topics, highlighting the role of specific medical treatments in predicting frailty.

**Conclusions:**

Integrating unstructured clinical notes and structured EHR data led to a notable enhancement in predicting frailty. This method shows great potential for standardizing frailty assessments using real-world data and improving patient selection for TAVR.

## Introduction

Degenerative severe aortic stenosis is estimated to affect 3.4% of the older population [1]. It results in reduced cardiac output, which reduces activity tolerance and often restricts the ability to perform activities of daily living [2,3]. This stage is associated with high short-term mortality [4]. The most common treatment that helps many patients resume an active lifestyle is transcatheter aortic valve replacement (TAVR), a less invasive procedure than open-heart surgery that replaces the diseased or damaged aortic valve [5]. Unfortunately, about 25% to 35% of patients either die or achieve no functional, morbidity, or mortality benefit from TAVR [6,7]. To optimize patient selection and postsurgical outcomes, accurate tools to acquire personalized, pertinent health data for TAVR candidates can provide valuable insights for patients and their clinicians to make informed decisions in presurgical clinics [6,8].

The American College of Cardiology’s guidelines [9], along with various clinical studies [8,10,11], have identified frailty as a risk factor for post-TAVR mortality and morbidity. In a systematic review, Sepehri et al [12] demonstrated the relationship between frailty and adverse postsurgical outcomes in the cardiac population. In addition, research in health economics has shown that frail patients undergoing cardiac procedures incur higher hospitalization costs than nonfrail patients [13]. Given the clinical and economic implications of the TAVR procedure, there is a need to standardize the frailty assessment. This can aid in formulating strategies to enhance post-TAVR outcomes in frail patients. Such strategies may include partnering with geriatricians, opting for monitored anesthesia care, and employing 2 surgeons for complicated procedures, among several others [14].

Existing risk scores, such as the Society of Thoracic Surgeons score and the European System for Cardiac Operative Risk Evaluation, are commonly used for patients referred for cardiac surgery. These scores are useful, but they are stratified into lower-risk patient groups referred for cardiac surgery and do not meet the specific needs of TAVR patients [15-17]. The current methods of assessing frailty in patients undergoing TAVR have significant limitations. A wide variety of tests and instruments are used across studies, many of which have not been robustly evaluated for this specific patient population [15,18]. For example, some studies use subjective methods such as the eyeball test, which relies on visual assessments to gauge frailty but lacks standardization and objective criteria [15]. Other instruments like the Katz Index of Independence in Activities of Daily Living [19] and the Kansas City Cardiomyopathy Questionnaire [20] are often employed in TAVR clinics to assess functional dependency in older individuals, focusing on daily living activities like feeding, bathing, and dressing. While these assessments are valuable in evaluating different aspects of frailty, they vary significantly in their focus and methodology, leading to inconsistencies in frailty classification among TAVR patients. Additionally, they are not specific to the TAVR population. The Fried frailty phenotype [21] and the Rockwood Frailty Index [22] are well-established and validated frailty assessments. However, these assessments require a detailed physical examination, patient interviews [21], and the completion of surveys [22], which may be time consuming and resource intensive. For example, the Fried frailty phenotype assessment takes 15‐20 minutes [23], and the Rockwood Frailty Index requires answering a 70-item questionnaire [22], which can burden health care providers and patients, hindering the adoption of frailty assessments and raising concerns about their feasibility, particularly in high-volume and resource-limited settings.

The goal of this study is to create a novel method that utilizes both structured and unstructured real-world data, along with machine learning (ML) techniques, to construct a frailty index that is specific to patients with aortic stenosis who are scheduled to undergo a TAVR procedure.

## Methods

### Ethical Considerations

This retrospective study utilized perioperative data collected from January 2018 to December 2019, as part of a federal study (R01 AG055337, CP) approved by the institutional review board of the University of Florida, with a waiver for Health Insurance Portability and Accountability Act compliance and an honest broker for medical record retrieval. The research adhered to the Declaration of Helsinki and followed all the institutional protocols. Participants provided written informed consent at the time of data collection.

### Study Population

The study extracted data on 14,000 patients from a deidentified historical database. A specific subset, the TAVR cohort, was identified using the Current Procedural Terminology code 33361, which narrowed the focus to 131 patients. All these patients had aortic stenosis as one of their symptoms.

### Frailty Phenotype and Target Variables

A person is classified as frail if they meet 3 or more of the following conditions: (1) unintentional weight loss of 10 pounds or more over the past 6 months; (2) a sense of persistent exhaustion, characterized by a moderate feeling that normal activities were strenuous over the past week or a moderate inability to initiate activities; (3) a reduced pace in walking, as assessed by a nurse; (4) diminished grip strength, determined by a gender-specific T-score below −2.5, based on the highest grip strength recorded across 3 attempts using a Jamar hydraulic hand dynamometer (Model J00105, Lafayette Instrument Europe); and (5) a self-reported decrease in physical activity, gauged by the Duke Activity Status Index [24]. The scoring range is from 0 to 5; individuals meeting fewer than 3 of these criteria are considered nonfrail. This frailty assessment is documented in electronic health records (EHRs) to assist in planning perioperative care. In the preoperative anesthesia clinic, both the nursing staff and anesthesia professionals undergo training to perform frailty assessments, guided by published criteria [21,25], alongside documenting educational background and conducting cognitive evaluations.

### Topic Modeling

Latent Dirichlet allocation (LDA) is a statistical model used in the field of natural language processing to uncover the hidden thematic structure in extensive collections of documents. We used LDA to generate 150 topics on clinical notes collected 60 days before surgery. A total of 543,520 clinical notes from 18,513 patients were collected. We removed preoperative evaluation notes that documented the results of the Fried frailty phenotype score to avoid information contamination. During data preprocessing, we found that some clinical notes contained similar information about the patients. To overcome this issue, we calculated the similarity between clinical notes that share the same NOTE_KEY using cosine distance and removed the notes with a similarity score greater than 80%. Special characters were removed, and all uppercase letters were converted to lowercase. Three types of words from the clinical notes were removed: (1) high-frequency words that were not closely related to medical symptoms, (2) words with a frequency less than 30, and (3) common stop words. We further performed stemming to reduce the words to their root form to simplify and standardize the vocabulary. After the data preprocessing, a total of 170,731 notes from 18,012 patients were included. Topic modeling was based on 50,000 randomly selected clinical notes. Topics of the remaining clinical notes were predicted and aggregated to the individual level to represent if the patients had a mention of a certain topic.

### Structured EHR Data

Preoperative attributes extracted from structured EHR data encompassed patient details available up to 6 months prior to their frailty evaluation. These included sociodemographic data (like age, gender, race, and years of education), medical history and severity (such as cancer history and the American Society of Anesthesiologists [ASA] physical status classification [26]), along with the latest biochemical markers typically recorded in preoperative settings (eg, hemoglobin and hematocrit levels).

Sociodemographic attributes captured information such as the patient’s age at frailty evaluation, gender, race, marital and employment status, and educational attainment. We divided age into 3 categories: under 70 years, between 70 and 79 years, and over 80 years. Education level was quantified by the total years of formal education [27], with each year of progression counted and repetitions not contributing to the total. For instance, completion of high school was marked as 12 years, a bachelor’s degree as 16 years, and so on. Medical history and acuity features included various medical conditions, the ASA physical status score, and the type of clinical encounter.

Medical conditions were binary indicators based on whether a patient had certain conditions, with the *International Classification of Diseases and Related Health Problems*, *Tenth Revision*, diagnosis codes condensed into Clinical Classifications Software categories for a more clinically relevant summarization. Conditions appearing in at least 10% of a broader cohort were selected as features for the ML models. The ASA score, ranging from 1 to 5, assessed patient health, with scores 1-2 indicating low-risk or mild systemic disease, and scores 3 and above signaling serious systemic illness.

Biochemical features included hematocrit, hemoglobin, and platelet count, with hemoglobin and hematocrit levels recommended for evaluation in older patients (65 years and above) undergoing significant surgery or those with a history of severe anemia undergoing minor surgery.

### Integration of Topic Features and Structured EHR Features

We manually reviewed topics generated from topic modeling and removed those that were not related to medical findings, resulting in 121 topic features in total. TAVR patients without clinical notes were removed (n=34), resulting in 97/131 patients in the training and evaluating ML models. Table S1 in Multimedia Appendix 1 compares the demographics between patients who were removed due to the absence of clinical notes and those who were used for modeling. After combining features generated from topic modeling and those from structured EHR data, we performed least absolute shrinkage and selection operator (LASSO) regression to reduce the feature space. Different parameters for regularization strength were examined, and the one that achieved the highest area under the receiver operating characteristic curve (AUC) for predicting frailty was selected for feature selection. We further applied Extreme Gradient Boosting (XGBoost) on features selected from LASSO to predict frailty. XGBoost is an ensemble learning method that uses decision tree frameworks. It creates models to improve the efficacy of preceding trees by diminishing errors through gradient descent. The resulting frailty index from XGBoost is a continuous score between 0 and 1, representing the probability of a patient being predicted as frail; high scores indicate a greater likelihood of frailty in TAVR patients.

**Figure 1.**
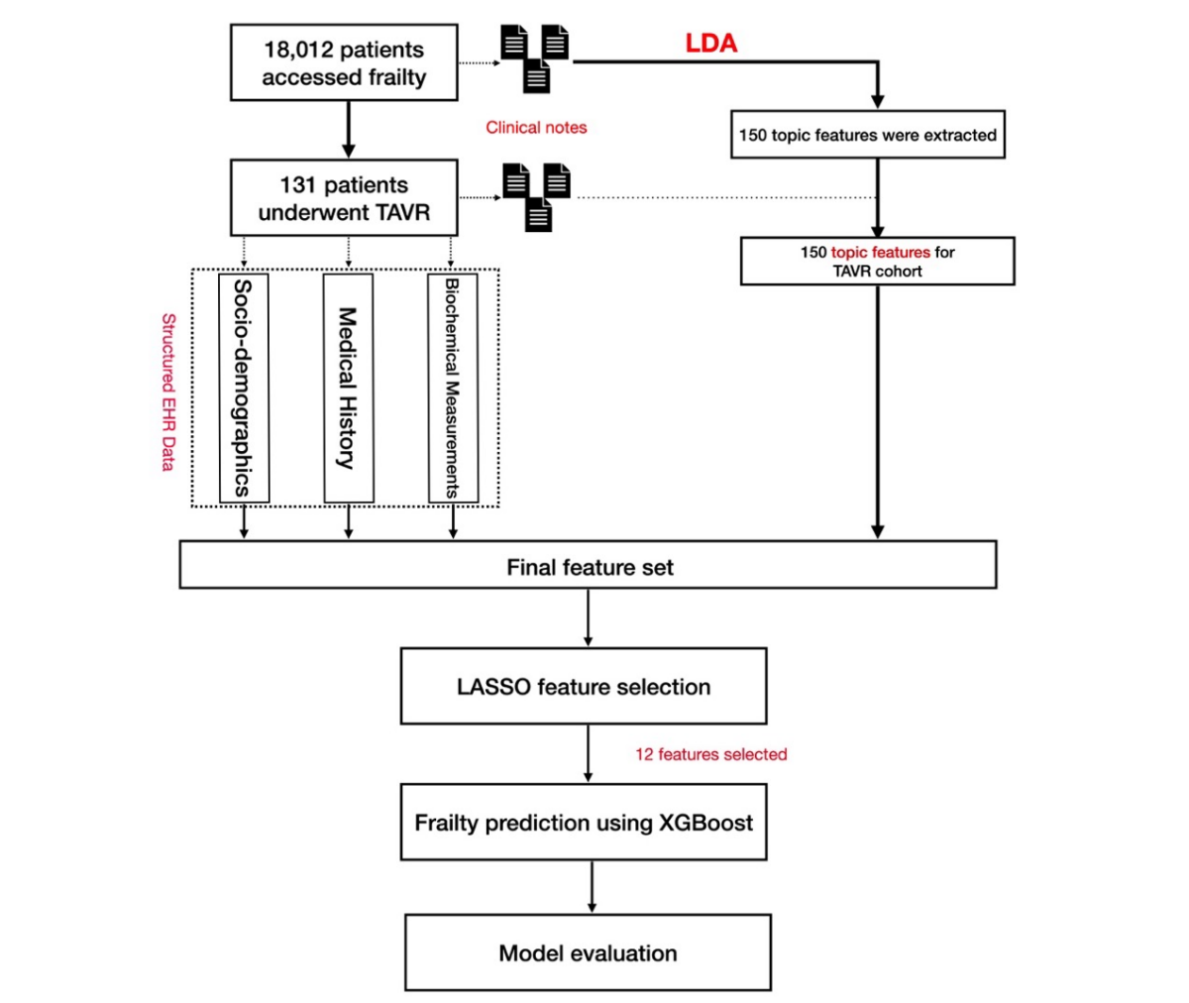
Overview of the analytic pipeline. EHR: electronic health record; LASSO: least absolute shrinkage and selection operator; LDA: latent Dirichlet allocation; TAVR: transcatheter aortic valve replacement; XGBoost: Extreme Gradient Boosting.

To assess the additive value of topic features derived from clinical notes, we also evaluated the performance of XGBoost on predicting frailty using only features from structured EHR data. We used the k-nearest neighbor imputation technique to impute missing values in numerical features with the mean value from the k-nearest neighbors (k=5) found in the training dataset. Missing values in categorical features were imputed as a new label (unknown). Numerical features were scaled to 0-1, and categorical features were encoded before training the ML model. Figure 1 illustrates the overview of the analytic pipeline.

### Model Performance and Evaluation

We employed a 5×5 nested cross-validation technique to evaluate our ML models. This technique consisted of 5 outer and 5 inner folds. During each outer fold, we set aside one-fifth of the patient records as an independent testing set, while the remaining four-fifths formed the training set. This outer training subset was then split into 5 inner folds for further validation. Each inner fold served as a stand-alone validation set, while the other 4 acted as the training set for the inner loop. The inner loop was responsible for training the models and fine-tuning the hyperparameters, taking a methodical approach in searching for the model’s ideal hyperparameter settings. Meanwhile, the outer loop estimated errors and evaluated generalization capabilities. To optimize hyperparameters, we utilized a grid search strategy to systematically explore various combinations of predefined hyperparameters to train our models. We calculated and reported the mean and SD for metrics like AUC, accuracy, sensitivity, and specificity across the 5 outer folds. This thorough approach boosts confidence in the models’ generalizability and scalability.

### Model Interpretation and Feature Ranking

We used the Shapley Additive Explanations (SHAP) method to explain how our trained ML models work. SHAP is a widely used model-agnostic explanatory approach that helps in understanding the outputs of ML models. We created a SHAP summary plot to demonstrate the importance of features and their impact on the outcome. This impact is shown through a sign and magnitude: the SHAP value’s sign indicates the direction of the feature’s impact on the outcome (eg, a positive SHAP value indicates that the feature in question increases the likelihood of frailty), while its magnitude reflects the feature’s predictive influence.

### Statistical Analysis

Categorical features between the frail and nonfrail cohorts were compared using a *χ*^*2*^ test for features with expected cell counts greater than 5 and a Fisher exact test for features with expected cell count less than or equal to 5 (ie, race). Age differences between the frail and nonfrail cohorts were compared using a Wilcoxson rank sum test. The significance level was set at *P*<.05.

## Results

Table 1 compares patient characteristics between frail and nonfrail patients who underwent TAVR. The most significant difference observed was that frail patients were older and had lower hematocrit and hemoglobin levels compared to nonfrail patients. However, there were no significant differences in other characteristics such as gender, race, marital status, education, ASA score, or employment status.

**Table 1.** Comparison of patient characteristics between frail and nonfrail TAVR^a^ patients.

Features	Frail (n=46)	Nonfrail (n=51)	*P* value
Age (years), mean (SD)	80.22 (7.45)	77.31 (6.05)	.04
Hematocrit (%), mean (SD)	36.16 (5.38)	39.93 (4.99)	<.001
Hemoglobin (g/DL), mean (SD)	12.14 (1.89)	13.4 (1.78)	.002
Platelet count (platelets/mL), mean (SD)	199.95 (86.34)	202.12 (51.91)	.88
Gender, n (%)			.69
Female (n=37)	19 (51.4)	18 (48.6)	
Male (n=60)	27 (45)	33 (55)	
Race, n (%)			.54
White (n=93)	43 (46.2)	50 (53.8)	
Others (n=4)	3 (75)	1 (25)	
Marital status, n (%)			.32
Married (n=65)	28 (43.1)	37 (56.9)	
Others (n=32)	18 (56.3)	14 (43.7)	
Years of education, n (%)			.41
Greater than 12 (n=58)	25 (43.1)	33 (56.9)	
Less than or equal to 12 (n=49)	21 (53.8)	28 (46.2)	
ASA^b^ physical status score, n (%)			.47
Less than or equal to 3 (n=21)	8 (38.1)	13 (61.9)	
Greater than 3 (n=76)	38 (50)	38 (50)	
Employment status, n (%)			.79
Retired (n=76)	35 (46.1)	41 (53.9)	
Not retired (n=21)	11 (52.4)	10 (47.6)	

a TAVR: transcatheter aortic valve replacement.

b ASA: American Society of Anesthesiologists.

Table S2 in Multimedia Appendix 1 shows the number of features selected by LASSO alongside corresponding performance metrics across different regularization parameter values. Lower regularization parameter values (ie, inversed regularization strength) correspond to increased penalization of coefficient magnitudes and reduced features retained in the final model. Specifically, an inversed regularization strength of 0.25 imposed the highest penalty, leading to the smallest feature selection. Comparatively, an inversed regularization strength of 0.5 resulted in 12 selected features and achieved the highest AUC (0.67) and accuracy (0.65).

**Figure 2.**
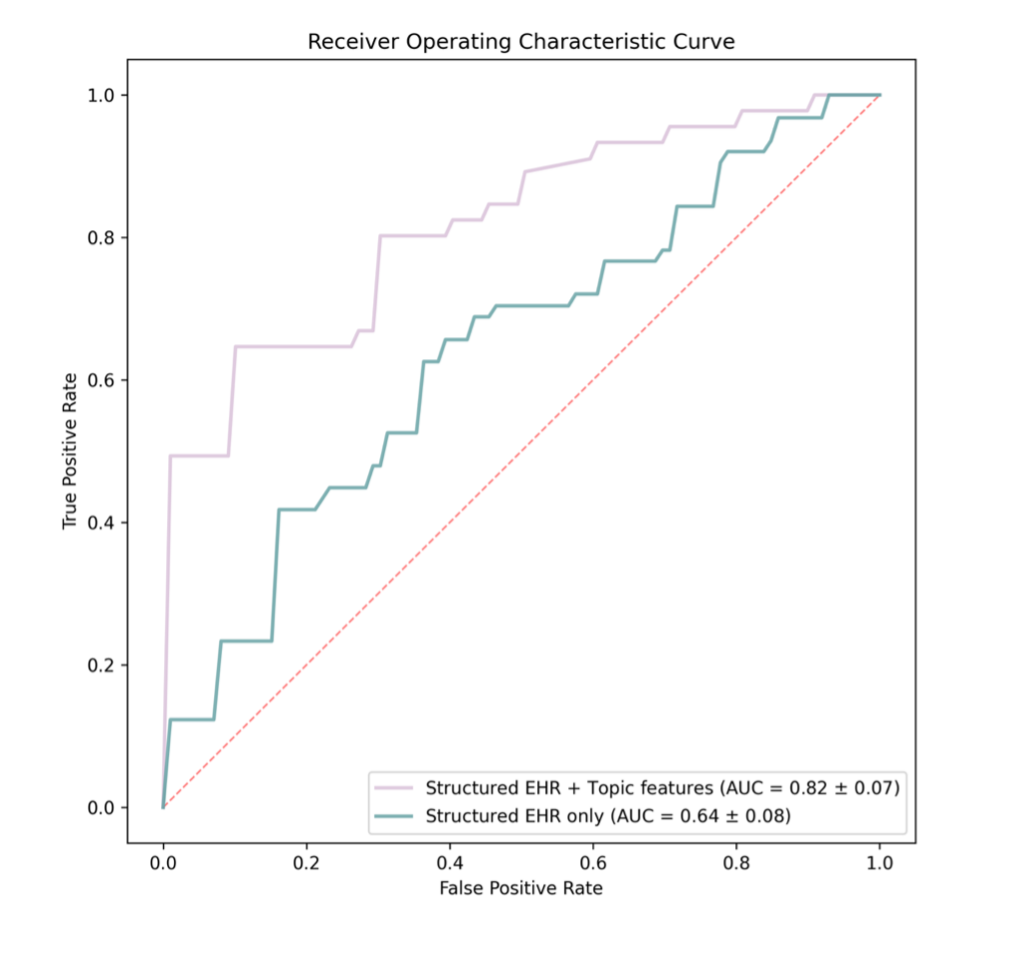
Receiver operating characteristic curves of frailty prediction using structured EHR data only and an integration of structured EHR data and topic features. Structured EHR features included sociodemographics, medical history and severity, and biochemical measurements. Topics features included features derived from clinical notes using latent Dirichlet allocation. The final features used were the ones selected from least absolute shrinkage and selection operator logistic regression. AUC: area under the receiver operating characteristic curve; EHR: electronic health record.

Table S3 in Multimedia Appendix 1 and Figure 2 show the performance of the ML models in identifying frailty. Results showed significant enhancement in the predictive performance when topic features derived from clinical notes were integrated with structured EHR data, compared to when only structured EHR data were used. Specifically, the combined topic + EHR model demonstrated a superior AUC of 0.82 (SD 0.07), indicating a robust ability to distinguish between frail and nonfrail individuals. This was a notable improvement over the EHR-only model, which achieved an AUC of 0.64 (SD 0.08).

**Figure 3.**
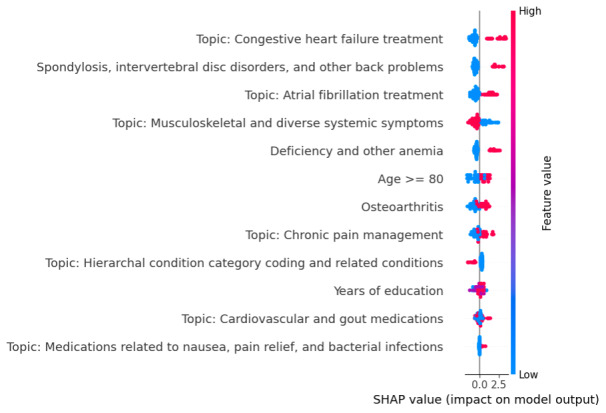
The rank of features in predicting the frailty phenotype. SHAP values greater than 0 (towards the right) contributed to predicting frailty, whereas SHAP values less than 0 contributed to predicting nonfrailty. For diagnosis and topic features, red dots indicate the presence, while blue dots indicate the absence of the condition. Red dots in “Years of education” represent patients with less than 12 years of education. Red dots in “Age >= 80” represent patients aged over 80 years old. SHAP: Shapley Additive Explanations.

Figure 3 shows the rank of features in predicting the frailty phenotype based on their SHAP values. Notably, the treatment and management of congestive heart failure (CHF) with carvedilol, spondylosis, intervertebral disc disorders, and other back problems and the treatment and management of atrial fibrillation (AFib) were found to have the strongest influence. These features exhibited high SHAP values, suggesting they strongly predict the frailty phenotype. Other significant factors included musculoskeletal symptoms, anemia, and age ≥80 years.

Table 2 presents 7 topics identified through LDA of clinical notes and further selected by LASSO. Of these, 5 topics related to the treatment and management of specific medical comorbidities in the cohort. The LDA algorithm identified underlying topics by clustering co-occurring terms across the clinical corpus. Each topic’s top 10 weighted terms indicated which terms appeared most prominently within a given cluster. Higher term weights represented stronger associations between the term and the topic. For instance, the medications “Carvedilol” (term weight 0.097) and “Coreg” (term weight 0.094) had the highest weights within the first topic. Based on the prominence of these cardioprotective drugs, the topics were interpreted as medical treatment and management of CHF.

**Table 2.** Topics selected by least absolute shrinkage and selection operator regression for predicting the frailty phenotype in transcatheter aortic valve replacement patients, along with the top associated terms (stemmed) and their respective weights. The topics and terms listed are critical factors identified by the least absolute shrinkage and selection operator model that contribute to the prediction of frailty. The weights indicate the strength and direction of each term’s association with the frailty phenotype, providing insights into the underlying factors influencing patient outcomes.

Topics	Details
Topic: Congestive heart failure treatment	0.097*“carvedilol”+0.094*“coreg”+0.056*“chf”+0.056*“lasix”+0.034*“bumex”+0.034*“spironolacton”+0.032*“cardiomyopathi”+0.029*“meal”+0.029*“bp”+0.027*“aldacton”
Topic: Atrial fibrillation treatment	0.168*“eliqui”+0.071*“fib”+0.069*“apixaban”+0.052*“xarelto”+0.047*“diltiazem”+0.035*“rate”+0.029*“stop”+0.028*“sotalol”+0.027*“hold”+0.025*“cardiovers”
Topic: Musculoskeletal and diverse systemic symptoms	0.058*“movement”+0.044*“swell”+0.041*“joint”+0.039*“musculoskelet”+0.033*“respiratori”+0.029*“extra”+0.026*“mucou”+0.026*“jaundic”+0.025*“psychiatr”+0.025*“rate”
Topic: Chronic pain management	0.197*“everi”+0.131*“tramadol”+0.091*“ultram”+0.080*“acetaminophen”+0.051*“nightli”+0.045*“ointment”+0.043*“appli”+0.038*“norco”+0.034*“bactroban”+0.032*“mupirocin”
Topic: Hierarchal condition category coding and related conditions	0.498*“hcc”+0.412*“code”+0.020*“mellitu”+0.014*“problem”+0.011*“anemia”+0.010*“list”+0.004*“neuropathi”+0.004*“pvd”+0.003*“coronari”+0.002*“defici”
Topic: Cardiovascular and gout medications	0.105*“tartrat”+0.102*“lopressor”+0.065*“gout”+0.064*“allopurinol”+0.057*“atorvastatin”+0.049*“zyloprim”+0.047*“lipitor”+0.042*“male”+0.042*“nightli”+0.036*“physic”
Topic: Medications related to nausea, pain relief, and bacterial infections	0.210*“everi”+0.073*“zofran”+0.060*“ondansetron”+0.043*“acetaminophen”+0.038*“amoxicillin”+0.034*“releas”+0.027*“odt”+0.027*“keflex”+0.025*“augmentin”+0.024*“delay”

## Discussion

### Principal Findings

This study developed a frailty index using ML techniques by combining structured and unstructured real-world data. Incorporating the features obtained from unstructured clinical notes and structured EHR data significantly improved the model’s performance in identifying frailty. SHAP revealed that cardiovascular conditions, specifically CHF and AFib, as well as musculoskeletal disorders, were the primary factors that predict frailty among patients. This is consistent with the mainstream of clinical evidence [28-30]. The prominence of age as a predictor supports the well-established association between advancing age and increased vulnerability to frailty and limited quality of life improvement post TAVR [31]. The impact of years of formal education on frailty prediction was relatively lower (ranked 10th in importance), indicating that while socioeconomic factors contribute to health outcomes, clinical characteristics are more determinative in the context of frailty. This clinical ML application can easily be integrated into pre-TAVR settings to guide perioperative management, heart team discussions, and resource utilization. Our model provides actionable and semiquantitative insights and can potentially highlight targets for optimization (eg, anemia). In addition, the data processing and derivation pipeline we practically demonstrate allows ML model retraining to accommodate updates in practice and interinstitutional context differences.

Structured real-world data that contain rich historical and current information across various domains (eg, diagnoses, procedures, demographics) have been leveraged to predict frailty. Segal et al [32] constructed ML models using medical claims data to predict the Fried frailty phenotype, achieving an AUC of 0.76 with a gradient boosting machines algorithm. Similarly, Le Pogam et al’s [33] exploration using inpatient discharge data obtained an AUC of 0.71, while Bai et al [34] harnessed structured EHR data to predict preoperative frailty, achieving an AUC of 0.74 using XGBoost. Notably, a considerable amount of information contained within EHRs exists as unstructured text. Recent advancements in ML and natural language processing have enabled researchers to harness this valuable resource, which traditionally posed substantial challenges for manual analysis. For instance, Shao et al [35] used topic modeling to extract frailty-related topics from clinical notes and defined frailty using the number of frailty-related topics present. This frailty index was associated with a 2-fold increased risk of the composite outcome combining 1-year hospitalization and mortality. Further, Chen et al [36] and Martin et al [37] developed deep learning models to predict sentences indicating geriatric syndromes or actionable frailty aspects and achieved an *F*_1_-score of 0.61 and a scaled Brier score of 0.52. Despite these advances, a gap remains in the literature concerning the integration of structured and unstructured real-world data for frailty prediction. Moreover, the development of frailty indices tailored to preoperative contexts, especially for TAVR patients, remains unexplored.

Our results demonstrated that clinical notes serve as a complement to structured EHR data in predicting preoperative frailty. Incorporating features extracted from clinical notes significantly improved the model’s performance over using only structured EHR data, with the integrated model using only 12 features achieving an AUC of 0.82 compared to 0.64 for the EHR-only model in the test set. This performance is superior to the one-size-fits-all statistical models such as the Society of Thoracic Surgeons score, which has an AUC of 0.64-0.7 in risk stratifying patients [38-40].

Identifying the key topics and associated terms provides actionable insights into understanding and managing frailty-associated factors. For instance, knowing that the patient has heart failure requiring cardiac medications and contributing to frailty may trigger putting the patient on a more intensive pre- and post-TAVR optimization pathway, such as right heart catheterization, to optimize heart failure therapy preprocedures. Similar approaches can be applied to other cardiovascular conditions or musculoskeletal disorders that are partially correctable with focused pre- or postprocedural conditioning. The topic of CHF treatment and management, with high-weight terms like “Carvedilol,” can highlight high systemic vascular resistance hemodynamic phenotypes, systolic heart failure, or arrhythmia requiring beta blockade. Similarly, the topic focusing on AFib management, with terms like “Eliquis” and “Apixaban,” highlights the need to evaluate and manage arrhythmias, which are risk factors for stroke and also are associated with obesity, hypoventilation, and chronically low physical and cardiopulmonary conditioning. The specificity of terms and their weights in each topic provides a framework for health care professionals to identify and prioritize areas of concern in pre-TAVR patients, ensuring a comprehensive and tailored approach to frailty assessment. Identifying “high-risk candidates” is crucial in determining a patient’s readiness for TAVR and optimizing postprocedural outcomes by addressing the multifaceted aspects of frailty and medical complexity in this vulnerable patient population [41,42]. A considerable advantage to using ML models is that they are translatable to new TAVR techniques, adaptable to clinical settings and automated facilitating implementation, and updatable to accommodate shifts in populations and practices.

Compared to the traditional frailty measurements, a key advantage of our developed method is its applicability to newly admitted patients with severe aortic stenosis being considered for TAVR. The model can process a patient’s available information, including both structured EHR data and unstructured clinical notes, to generate a frailty index score once the patient is admitted. This assessment provides an objective measure of a patient’s frailty status, which has the potential to enhance the clinical shared decision-making between physicians and the patient. By offering an early and timely indication of a patient’s frailty status, this approach allows for a more informed discussion about the risks and benefits of TAVR, potential alternative treatments, and the need for additional preoperative interventions and postoperative care plans, ultimately improving the care of TAVR patients.

This study, while insightful, acknowledges specific limitations. It utilizes retrospective EHR data, a method that provides a valuable foundation for analysis despite its inherent limitations, such as the potential for minor reporting inconsistencies, recall variations, and lead time considerations. The study’s focus on frailty, although a topic of ongoing discussion due to its varied definitions and intersection with medical complexity, enriches the discourse by offering a nuanced view of its implications. Additionally, while our ML approach effectively integrates structured and unstructured real-world data to predict frailty, it does not extensively address potential confounding factors such as comorbidities, medications, or socioeconomic status. This focus on predictive modeling, rather than causal analysis, may limit the ability to fully account for these variables. Furthermore, this study has not undergone external validation; however, this aspect is mitigated by the rigorous internal review processes, which reduce the risk of overfitting and ensure the model’s preliminary reliability. Rather than significantly limiting, these considerations underscore the study’s contribution to ongoing research and its potential as a springboard for further investigation and external validation efforts.

### Conclusion

This study demonstrates the effectiveness of combining unstructured clinical notes with structured EHR data to assess frailty in TAVR patients. This approach can potentially improve risk stratification for TAVR, adding insight into targets for perioperative optimization and potentially improving TAVR outcomes. Future efforts should integrate these models into clinical settings to improve TAVR planning and patient care, emphasizing continuous medical education and innovation.

## Supplementary material

10.2196/58980 Multimedia Appendix 1 Supplementary material.

## Abbreviations

AFib: atrial fibrillation
ASA: American Society of Anesthesiologists
AUC: area under the receiver operating characteristic curve
CHF: congestive heart failure
EHR: electronic health record
LASSO: least absolute shrinkage and selection operator
LDA: latent Dirichlet allocation
ML: machine learning
SHAP: Shapley Additive Explanations
TAVR: transcatheter aortic valve replacement
XGBoost: Extreme Gradient Boosting
