# Supplementary material for: Enhancing Frailty Assessments for Transcatheter Aortic Valve Replacement Patients Using Structured and Unstructured Data: Real-World Evidence Study
Source: JMIR Aging. 2024 Nov 27;7:e58980. doi: 10.2196/58980 (PMC11612520; doi:10.2196/58980)
Supplement: Multimedia Appendix 1 [file aging-v7-e58980-s001.docx]

**Supplementary Material**

**Supplementary Table S1.** Comparison of demographics between patients who were removed due to the absence of clinical notes and those who were used for modeling.

|  | Patients without clinical notes (N=34) | Patients with clinical notes  (N=97) | p-value |
| --- | --- | --- | --- |
| Age, mean (SD), years | 80.0 (8.1) | 78.7 (6.9) | 0.37 |
| **Frailty, No. (%)** |  |  | 0.72 |
| Yes | 18 (52.9%) | 46 (47.4%) |  |
| No | 16 (47.1%) | 51 (52.6%) |  |
| **Gender** |  |  | 0.91 |
| Female | 14 (41.2%) | 37 (38.1%) |  |
| Male | 20 (58.8% | 60 (61.9%) |  |
| **Race, No. (%)** |  |  | 0.53 |
| White | 34 (100%) | 93 (95.9%) |  |
| Others | 0 (0.00%) | 4 (4.1%) |  |
| **Marital Status, No. (%)** |  |  | 0.002 |
| Married | 12 (35.3%) | 65 (67.0%) |  |
| Others | 22 (64.7%) | 32 (33.0%) |  |
| **Education Years, No. (%)** |  |  | 0.25 |
| Greater than 12 | 14 (50.0%) | 58 (64.4%) |  |
| Less than or equal to 12 | 14 (50.0%) | 32 (35.6%) |  |
| **ASA Physical Status Score, No. (%)** |  |  | 0.53 |
| Greater than 3 | 29 (85.3%) | 76 (78.4%) |  |
| Less than or equal to 3 | 5 (14.7%) | 21 (21.6%) |  |
| **Employment Status, No. (%)** |  |  | 1.0 |
| Retired | 26 (76.5%) | 76 (78.4%) |  |
| Not retired | 8 (23.5%) | 21 (21.6%) |  |

**Supplementary Table S2**. Comparison of inversed regularization strength of LASSO on the performance of feature selection.

| **Inversed regularization strength (C)** | **Number of selected features** | **AUC** | **Accuracy** |
| --- | --- | --- | --- |
| 1.0 | 33 | 0.63 | 0.55 |
| 0.75 | 26 | 0.65 | 0.60 |
| **0.50** | **12** | **0.67** | **0.65** |
| 0.25 | 3 | 0.61 | 0.60 |

**Supplementary Table S3**. The predictive performance of machine learning models was trained using features derived from clinical notes and only structured EHR features.

| **Model** | **AUC** | **Accuracy** | **Sensitivity** | **Specificity** |
| --- | --- | --- | --- | --- |
| Topic+EHR | 0.82 (0.07) | 0.73 (0.09) | 0.69 (0.17) | 0.77 (0.19) |
| EHR only | 0.64 (0.08) | 0.64 (0.11) | 0.67 (0.10) | 0.61 (0.21) |
